# Object-Oriented and Visual-Based Localization in Urban Environments

**DOI:** 10.3390/s24062014

**Published:** 2024-03-21

**Authors:** Bo-Lung Tsai, Kwei-Jay Lin

**Affiliations:** 1 Department of Electrical Engineering and Computer Science, University of California, Irvine, CA 92697, USA; klin@uci.edu; 2 College of Intelligent Computing, Chang Gung University, Taoyuan 333, Taiwan

**Keywords:** visual-based localization, pose estimation, object detection, Internet of Things

## Abstract

In visual-based localization, prior research falls short in addressing challenges for the Internet of Things with limited computational resources. The dominant state-of-the-art models are based on separate feature extractors and descriptors without consideration of the constraints of small hardware, the issue of inconsistent image scale, or the presence of multi-objects. We introduce “OOPose”, a real-time object-oriented pose estimation framework that leverages dense features from off-the-shelf object detection neural networks. It balances between pixel-matching accuracy and processing speed, enhancing overall performance. When input images share a comparable set of features, their matching accuracy is substantially heightened, while the reduction in image size facilitates faster processing but may compromise accuracy. OOPose resizes both the original library and cropped query object images to a width of 416 pixels. This adjustment results in a 2.4-fold improvement in pose accuracy and an 8.6-fold increase in processing speed. Moreover, OOPose eliminates the need for traditional sparse point extraction and description processes by capitalizing on dense network backbone features and selecting the detected query objects and sources of object library images, ensuring not only 1.3 times more accurate results but also three times greater stability compared to real-time sparse ORB matching algorithms. Beyond enhancements, we demonstrated the feasibility of OOPose in an autonomous mobile robot, enabling self-localization with a single camera at 10 FPS on a single CPU. It proves the cost-effectiveness and real-world applicability of OOPose for small embedded devices, setting the stage for potential markets and providing end-users with distinct advantages.

## 1. Introduction

Real-time and precise localization is a key technology for autonomous robotics deployed in many Internet of Things (IoT) applications like residential health assistants [1], delivery services [2], commercial guides [3], augmented industrial training [4], or metaverse [5]. To enable these smart services, they must be capable of sensing the surrounding spatial context and maintaining the consistency with which digital content reacts and aligns with the physical environment. Visual landmarks are all-around in modern cities and indoor areas, and visual-based localization (VBL) [6] is one of the rising localization techniques that are accurate, reliable, and economical in urban environments like narrow streets and near-building pedestrian walkways where GPS or wireless signals are prone to interference or blocked by walls. With only cameras, VBL makes IoT autonomous devices available and convenient to empower human-robot interaction with location-aware capability in those intelligent services. To locate a camera, VBL studies adopt map models and establish the spatial relationship between the input image pixels and the map. The underlying map models can be categorized into two types: direct images and indirect local features extracted from the images. There are trade-offs between model complexity and service quality. The more details a model can handle, the higher the location service quality, but the greater the burden on processing efficiency. In the next two sections, we explore different map models used in visual-based localization and compare direct and indirect map models to understand their strengths and weaknesses. Particularly, we highlight the object-based model, an improvement of the indirect map model with practical advantages. In addition, we address current challenges with those methods, providing insights into ongoing issues in the field.

### 1.1. Background

For the direct image model, VBL searches and retrieves similar images by comparing global descriptors or neural network features, triangulating positions from geo-tags [7]. It approximates camera locations with meter-level errors due to label precision and image comparison granularity, making it suitable for applications like large-scale place recognition or scenic tours where a rough location suffices.

Researchers focus on indirect feature-based methods to reduce errors to centimeters, ideal for urban navigation indoors or outdoors. The map model stores features and positions, estimating camera pose from matched features between input and map images. It calculates location by matching 2D features at pixel-level or semantic object-level. Classical VBL matches 2D pixel-level sparse features between query and database images for higher accuracy [8], but high-end GPU runtime costs are impractical for IoT devices [9].

**Figure 1:**
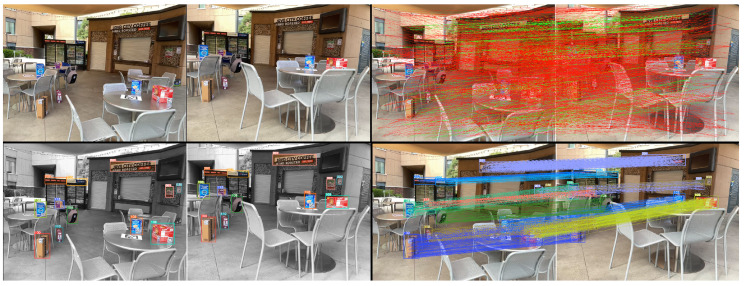
Comparison of the standard matching and our object-oriented matching approach on whole images. (**Top Left**): The picture on the left is the reference frame, and the right is the query frame captured by a robot camera at the Javacity Cafe. (**Top Right**): The standard ORB real-time matching from the reference to query. (**Bottom Left**): The unique objects are detected by Yolov5 on both frames with bounding boxes in various colors. (**Bottom Right**): Our object-oriented compact feature matches from reference to query frame by reusing features stored in object detectors.

Exhaustive matching of detected pixel-based features in full images is inefficient. Real scenes often contain competing patterns, causing feature descriptor mismatches. In Figure 1, top-left dual photos show reference and query frames in a cafe environment, with top-right dual photos displaying brute force matching results from ORBSLAM3 [10]. Only 20.4% of matched feature points fell within the correct region, indicating mismatches due to viewing angle changes and new feature points.

Objects in the environment provide fixed areas for feature matching, with absolute 3D models and known sizes, reducing matching uncertainty. With object detection advancements, objects serve as landmarks for faster and more accurate localization. Our framework isolates search space semantically, eliminating cross-region mismatches, as seen in the bottom row of Figure 1.

**Figure 2:**
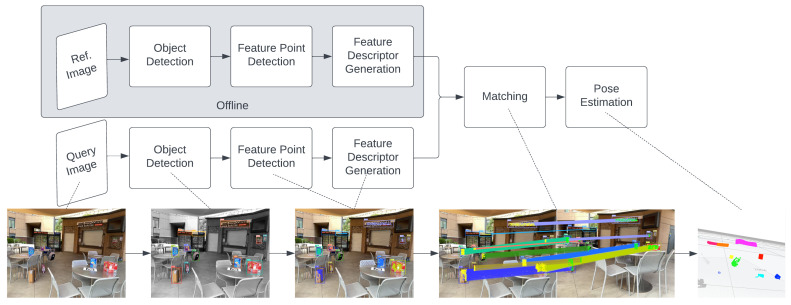
Standard object-based VBL pipeline: it consists of five stages, including object detection, feature point detection, feature description, feature matching, and the final pose estimation.

Object-based VBL involves several steps (as described in Figure 2): object detection, feature point detection, feature description, feature matching, and pose estimation [11]. When the camera captures an image, the object detector locates and identifies objects. Points within cropped object regions with high variance are detected, and feature descriptors are generated based on surrounding pixels. Both the query and reference images undergo these stages, with the reference image processed offline and its 3D locations collected. Matching algorithms pair descriptors with the closest distance in vector space, enabling camera location estimation from the correlated sets of points from 2D to 3D coordinates.

While artificial planar objects are common in indoor scenarios [11,12], daily environments feature more 3D objects. Extending methods to 3D objects entails training detectors on object faces, but this introduces challenges with data balancing and parameter tuning due to shared patterns among faces, impacting detection and matching precision. Utilizing existing object detectors for both object recognition and feature extraction avoids the need for object-face-specific training.

Table 1 compares various VBL methods: direct image matching, indirect feature matching, and object-based methods like ArUco, Picpose, and OOPose. Object-based methods offer higher precision at an accurate scale compared to direct or indirect methods. ArUco and Picpose rely on 2D planar markers, with ArUco markers being black-and-white blocks bordered by square lines, while Picpose landmarks can be any planar object with patterns. OOPose markers can be both 2D and 3D patterns. Despite longer processing times for OOPose and Picpose due to neural network detection, they enable real-time localization on IoT devices. Further performance details will be discussed in this paper.

Several unresolved issues impact the speed and accuracy of these techniques, including input image scale affecting matching performance, additional stages of point feature processing, and the number of matched objects, as highlighted in Table 1. These issues will be addressed in the following section.

### 1.2. Problems and Challenges

**Figure 3:**
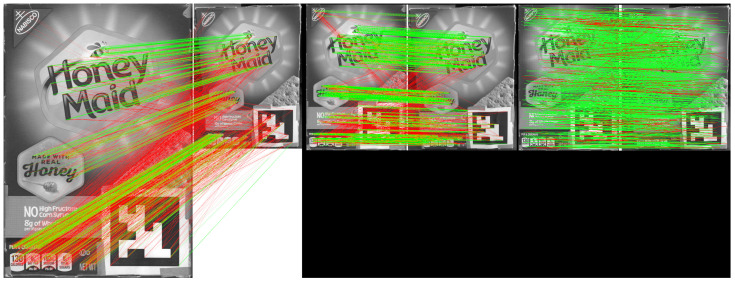
Comparison of standard matching and our object-oriented matching approach on object regions. (**Left**): The standard ORB real-time matching from the reference frame to the query frame. The sub-figure on the right is the reference image of the object model, and the left is the object region detected from a robot camera frame at the Javacity Cafe. (**Middle**): After adjusting the scale, the matching result of standard ORB matches from reference to query frame. (**Right**): With scale adjusted, Our object-oriented compact feature matches from reference to query frame by reusing features stored in object detectors.

One of the fundamental challenges to localization accuracy arises from scale differences between query and library reference images. Scale inconsistency occurs when regions cropped by the bounding box exhibit resolution disparities from the reference model image due to varying distances and viewing angles, leading to accuracy degradation and increased matching time. Matching performance depends on the similarity of the correct feature points. With similar scales, sparse feature points can be matched more accurately and quickly on object regions [14], significantly enhancing localization accuracy. As shown in Figure 3, 70% (1390 out of 2000 points) of ORB descriptors in the reference frame are erroneously matched to other points beyond a 10-pixel distance from the ground truth targets at original sizes, reduced to 33% (659 out of 2000) with proper scale adjustment. Our proposed object-oriented compact feature matching further reduces it to 17% (189 out of 1105). The next question pertains to determining appropriate sizes for both reference and query frames to optimize performance. While larger inputs potentially offer more accuracy due to clear and detailed patterns, they also incur greater computational costs. Resizing to smaller sizes may enhance speed but can blur pixels together, making features indistinguishable for matching, inevitably reducing accuracy. Balancing accuracy and computation time is crucial and will be discussed further.

Second, object detection, feature point detection, and feature descriptor generation all introduce delays to the process, suggesting benefits in combining these steps. Feature points can be detected, described, and matched without using information extracted during object detection. Dense feature maps in off-the-shelf object detection convolution layers provide the abstraction for image-matching tasks with a coarse-to-fine-based search on fixed neural network layers [15], achieving better matching accuracy without the need for further sparse feature point detection and description. While this leverages existing semantic features, it significantly increases computation time on low-end devices due to the dense map nature. Additionally, considering the deepest layer used for feature maps is 1/32 of the input size, setting the lower bound for region size and adjusting the input image size to be smaller than 128×128 pixels is impractical. However, dense features encompass all points, resulting in quadratic growth in computation to process and pair features with larger input sizes.

Third, much object-based localization research focuses on single-object pose estimation, but increasing the number of objects multiplies runtime and introduces noise to matching. Not all objects should be matched equally; library and query object regions should visually resemble each other for maximum accuracy. For instance, blurred object images are harder to extract and match features than sharp candidates, while aspect ratio indicates altered viewing angles or blocked sight. Carefully selecting objects for matching before feature matching can increase efficiency.

Last but not least, following object detection, retrieval of the 3D object library model for feature matching is necessary, unlike the planar model satisfied with single image models. This includes pre-built 3D models and localized 2D views of real-scene objects for higher matching and localization accuracy. For continuous and real-time localization, once objects are detected as landmarks in the first frame, it is unnecessary to redetect but instead track them in subsequent frames to save time and computational power.

**Figure 4:**
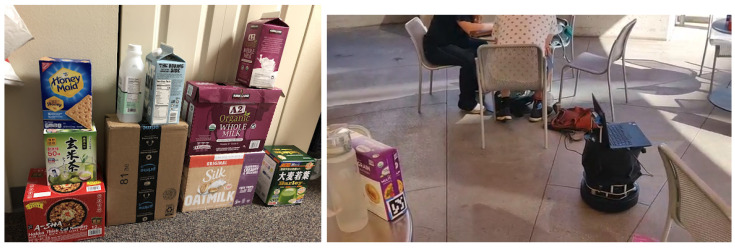
(**Left**): Examples of objects used (**Right**): Robot prototype equipped with the OOPose technology in the real world.

**Figure 5:**
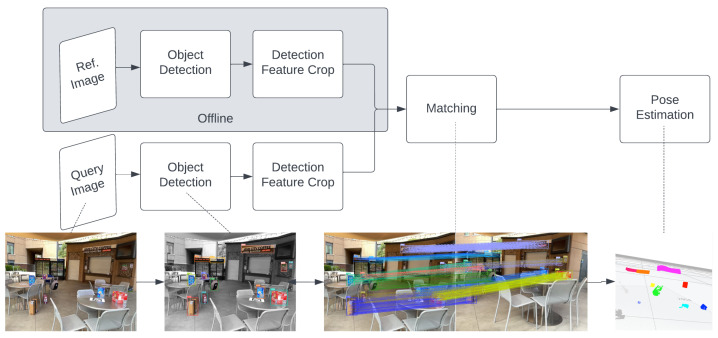
Our proposed object-oriented VBL pipeline: compared to the standard pipeline in Figure 2. It reduces the stages of feature point detection and description by reusing the semantic dense feature cropped from the object detection network.

This paper presents an object-oriented Visual-Based Localization (VBL) framework for providing 3D pose estimation using a camera in urban IoT applications. Figure 4 displays examples of objects on the left and a robot application utilizing our VBL technology on the right. The robot calculates its location based on pre-trained objects detected by the camera. Our method differs from the pictorial planar object VBL approach of Picpose [12], which relies on additional feature point descriptors of 2D planar objects. Instead, our framework extracts features from object detection and localizes a camera view by dynamically relating these features from the 2D object view to the corresponding features of the 3D model stored in the map. The proposed pipeline is depicted in Figure 5, eliminating the need for unnecessary feature point detection and descriptor generation, as these are fulfilled during the object detection stage.

The contribution of our VBL method can be summarized as follows:

- A low-cost localization method using a single camera and mobile CPU/GPU for IoT applications.
- Reusing off-the-shelf object detector features dynamically at appropriate scales for accurate, faster, and robust pose estimation without requiring an additional network or point feature detector.
- Flexibility to handle not only planar pictures but also daily 3D objects without CAD models, suitable for complex urban indoor and outdoor environments.
- Selective pose estimation from either the library or actual scene object models for real-time performance, offering a practical solution for IoT localization.

The remainder of the paper is organized as follows: In Section 2, we review background information related to visual-based localization. Section 3 describes the design of our object-oriented localization framework and provides an overview of its major components. Section 3.2.1, Section 3.2.2 and Section 3.2.3 present the design and performance of DynaScale2, our compact feature matching technique, and object selection strategies, respectively, addressing the identified issues. Section 4 presents studies and performance statistics collected from our robot prototype. Finally, Section 5 concludes the paper.

## 2. Related Work

This paper aims to propose an object-oriented feature-based visual localization method, and in the following sections, we review relevant techniques and methods from the literature.

### 2.1. Visual Feature-Matching Based Localization

Visual-based localization (VBL) has been extensively studied with feature-matching algorithms. The goal is to establish 2D-3D correspondences between visual feature descriptors extracted from the 2D input image and those in a pre-existing 3D model obtained through the structure from motion (SfM) [16]. Various local features have been explored, including statistical-based SIFT [17], RootSIFT [18], binary-based FAST [19], ORB [20], learning-based LIFT [21], D2Net [22], and SuperPoint [23]. Matching typically involves comparing feature vector correlation, applying mutual nearest neighbor ratio tests [17], or employing learned attention mechanisms like SuperGlue [24]. Research efforts have focused on enhancing the efficiency and robustness of both feature descriptors [25,26,27,28,29,30,31,32] and matching algorithms [33,34,35,36,37,38] to cope with varying environmental conditions that can affect performance [39].

In addition to sparse point matching, dense matching involves finding correspondences across all points on feature maps. Methods like dense SIFT descriptors [40] and direct matching within pre-trained neural networks such as NCNet [41] have been explored. While dense feature points offer high robustness and accuracy, exhaustive matching on entire images leads to slower speeds. Techniques like adapting feature extraction layers from object detection networks to produce semantic dense feature maps and matching from sparse to dense targets [42], or matching from deep to shallow layers [15], have been proposed to mitigate this issue. However, even feature-based methods encounter challenges in large-scale scenes due to the proliferation of local features [43].

To address scalability, image databases with global representations of pose-annotated scene images are utilized for retrieving top-ranked related images for feature matching. Global features like VLAD [44] and its learning-based variant NetVLAD [45] aggregate local features into compact representations for database queries. This retrieval step enables feature-matching methods to be applied in large-scale environments by first obtaining coarse locations and refining them for more accurate pose estimation without matching the entire feature set.

However, the accuracy and speed of localization are influenced by input quality, feature encoding, and computation size. Environmental changes, illumination variations, or shifts in viewing angles pose challenges for feature descriptors. Even learning-based features may not generalize well across different datasets [8]. Moreover, the image retrieval step in hierarchical localization is computationally expensive for real-time performance on small devices, necessitating additional global feature processing on high-end GPUs [8].

### 2.2. Visual Object Based Localization

With the advancement of object detection, semantic information plays a significant role, as these features are more invariant to challenging conditions, enhancing localization performance in matching or pose estimation techniques [46,47,48,49,50]. Visual object-based localization generally encompasses two types similar to VBL: model-based and model-free.

Model-based methods rely on accurate CAD models to either directly regress pose from features within the region of interest [51,52,53,54] or find correspondences between 2D pixels and 3D object models through regression techniques [55,56,57]. While these methods typically require a separate network for each instance, NOCS [58] eliminates this need during online testing by extracting normalized object coordinates for each category of objects.

In contrast, model-free methods abandon accurate CAD models. Early methods of this kind attempted to synthesize images for matching by constructing latent spaces [59] or regressing corner coordinates of objects for each category with extensive annotated training data [60]. However, these methods pose challenges for IoT devices due to the additional computation of instance-specific or category-specific networks or the need for a significant amount of training data annotations. On the other hand, PicPose utilizes planar object detection, ORB feature extraction, and matching on objects to achieve real-time localization on generic objects. OnePose is the first method to eliminate the need for annotations and category-specific models by registering the object model online and directly matching 2D points to 3D models using a learned attention network [61]. It exhibits faster performance than HLoc [8], although it does not consider the time cost of detection nor reuse the feature detection network.

### 2.3. Visual Feature Fusion Techniques

Regarding semantic feature fusion, various techniques exist, including attention-based networks [62,63], generative adversarial networks [64], self-supervised learning networks [65], weighted wavelet components [66], knowledge distillation [67], composition with other learned features [68], cascades of explicit models [69], and temporal optimization [70]. While these methods significantly improve original models in terms of precision or efficiency in various research domains, such as super-resolution [63], wheat classification [64], and noisy image enhancement [66], applying these strategies to real-time visual-based localization remains challenging due to the gaps between domains. Explicit features are necessary for precise and robust matching, and minimizing time costs requires fewer computation stages and the reuse of the produced intermediate features. Inspired by S2DNet [42] and DFM [15], we extend dense features extracted from object detection networks and match them based on deeper and smaller features, searching layer-wise for nearest neighbors’ similarity.

## 3. Model Architecture

### 3.1. Object-Oriented Visual-Based Localization

The essence of object-oriented localization lies in detecting unique objects within an area and recognizing various sets of objects in different scenes. However, in reality, it is impractical to learn every unique object globally due to the continuous creation of objects across space and time, as well as limitations in the detection model’s memory capacity. Therefore, uniqueness is constrained within a certain range, where an object does not duplicate with another of the same type. The smaller this range, the less unique the object is perceived. For a robot, the ability to differentiate between objects specific to its initial location is crucial, necessitating swift adaptation to distinct environments.

Object detectors categorize objects based on predefined classes established in the training dataset. They typically comprise two interconnected network architectures: a feature extraction backbone and a classification convolutional or fully connected network. During the offline training phase, modern object detectors leverage diverse datasets like COCO [71] or ImageNet [72] to encode feature representations through layers of configurable filters. Subsequently, these features are decoded to determine each object’s class and bounding region. Transfer learning techniques allow for the application of knowledge gained from object feature encoding to other tasks within the same domain. Consequently, parameters from the same classifier can be interchanged to detect different objects in various locations, while the feature extraction backbone can be reused for both detection and localization matching tasks.

Let *I* be a two-dimensional gray-scale image of size W×H, where W∈N is the width and H∈N is the height. I(u,v) is the image pixel value at the point location $p=\left(u,v\right)\in N^{2}$ where 0≤u≤W and 0≤v≤H. After object detection, it will output the bounding boxes of the objects with their IDs and confidence scores. ${Obj}_{i}=\left(u_{i}^{tl},v_{i}^{tl},u_{i}^{br},v_{i}^{br},{cls}_{i},{conf}_{i}\right)$, where $\left(u_{i}^{tl},v_{i}^{tl}\right)$, and $\left(u_{i}^{br},v_{i}^{br}\right)$ are the pixel locations of the top-left corner and bottom-right corner of the bounding boxes on image *I*, respectively, and ${cls}_{i}$ and ${conf}_{i}$ are the class ID and confidence score of a detected object ${Obj}_{i}$. During the forward propagation of the neural network detector $f:R^{W\times H}\to R^{|{Obj}_{i}|\times 6}$, we will retain the output of the intermediate layers as the dense features. For a neural network with an input image *I*, $f\left(I\right)=f_{K}\circ f_{K-1}\circ \cdots \circ f_{2}\circ f_{1}\left(I\right)$ where $f_{k}$(1≤k≤K) is the operator functions, including convolution functions, bottleneck functions, pooling functions, fully connected functions, or activation functions [73,74,75]. Some operator functions result in the down-sampled intermediate output (commonly half the size of the width and height of the immediate previous function input), where $f_{k}\left(F_{k}\right)=F_{k+1}:R^{W_{k}\times H_{k}\times Ch_{k}}\to R^{W_{k+1}\times H_{k+1}\times Ch_{k+1}}$ and $W_{k}=2\times W_{k+1}$, and $H_{k}=2\times H_{k+1}$. $F_{k}$ and $F_{k+1}$ are the feature map input and output of the function $f_{k}$, respectively. $Ch_{k}$ and $Ch_{k+1}$ are the depth (or channels) of input and output in bits, respectively.

Most competitive models have additional feature point extraction and description stages right after object detection and then use those point features to match against the ones stored in the library. The pose can be estimated from the matching pairs among all the detected objects. Here, for each object region on the image, ${Obj}_{i}\in \left\{{Obj}_{i}\right\}$, those models extract from the object region a set of keypoints $\left\{p_{key}^{i}\right\}$ and corresponding descriptor set for matching $\left\{d_{key}^{i}\right\}$, as in the following two formulas, FeatureExtraction and FeatureDescription, which are the two stages of feature point extraction and feature vector description:

(1) $$\mathrm{FeatureExtraction}\left(I,\left\{{Obj}_{i}\right\}\right)=\left\{p_{key}^{i}\right\}$$

(2) $$\mathrm{FeatureDescription}\left(I,\left\{p_{key}^{i}\right\}\right)=\left\{d_{key}^{i}\right\}$$

Given the feature points and descriptors of each query object, $\left\{p_{key}^{q}\right\},\left\{d_{key}^{q}\right\}$ as well as those points and descriptors of the library object with the same class ID, $\left\{p_{key}^{l}\right\},\left\{d_{key}^{l}\right\}$, a feature matcher function, Matcher generates a set of putative correspondences set, $\left\{\left({\hat{p}}_{key}^{l},{\hat{p}}_{key}^{q}\right)\right\}$, by comparing the similarities of the input descriptors as the following equation:

(3) $$\mathrm{Matcher}\left(\left\{p_{key}^{l}\right\},\left\{d_{key}^{l}\right\},\left\{p_{key}^{q}\right\},\left\{d_{key}^{q}\right\}\right)=\left\{\left({\hat{p}}_{key}^{l},{\hat{p}}_{key}^{q}\right)\right\}$$

Each 2D feature point p=(u,v) has its corresponding 3D point $x_{r}=\left(x_{r},y_{r},z_{r}\right)\in R^{3}$ of the object 3D models. The object 2D reference points are commonly the outer corner points of the objects, with one point being the origin of the local coordinate system. The 3D object map keeps the global 3D coordinates $x_{w}=\left(x_{w},y_{w},z_{w}\right)\in R^{3}$ of the reference points of all objects in a well-established world coordinate system such as ISO standard geographical maps.

A pose is defined by a given 3D world where its coordinate is naturally the motion from the origin of the world coordinate system. In the same way, either the mapping between the camera coordinate and another coordinate system or the camera movement, which is also a mapping between the old and new camera coordinates, can all be regarded as a Euclidean transform from one coordinate system to another. As long as the mapped coordinate system is established, the camera pose can be acquired from the reverse of the transform. This transform matrix, T, is composed of six degrees of freedom (6DoF): forward/back, up/down, left/right, yaw, pitch, and roll. The first three degrees of freedom constitute the translation movement, $t\in R^{3}$, while the rest describe the rotation, $R\in R^{3\times 3}$. A motion is then expressed as the following equation from one 3D coordinate $x_{w}^{1}$ to another destination coordinate $x_{w}^{2}$:

$$x_{w}^{2}=R\cdot x_{w}^{1}+t=T\cdot {\left[x_{w}^{1},1\right]}^{T}\mathrm{where}\;T=\left[\begin{array}{cc} R & t \end{array}\right]\in R^{3\times 4}$$

The projection of a 3D coordinate onto the pinhole camera’s 2D pixel plane involves a series of matrix multiplications: $p=s\cdot K\cdot {\left[x_{w}^{2},1\right]}^{T}=s\cdot K\cdot T\cdot {\left[x_{w}^{1},1\right]}^{T}$ where $K\in R^{3}$ is the camera intrinsic projection matrix calibrated with the focal lengths and the camera optic center pixel location, and s∈R is the scale factor to measure the depth distance in the unit of the focal length inferred from equiangular triangles between the camera, camera focus, and the object. With the matching of pixels between the library object model, $p^{l}$, and the camera query frame, $p^{q}$, we could derive the matrix *T* from the equation.

The OOPose follows the overall architecture, but it does not need additional feature detection and description because of the abundant dense semantic features already extracted during object detection, which are the feature layers $F_{k}$. So before the matching, it could directly crop from those dense features F based on the object region, as in the following equation, FeatureCrop:

(4) $$\mathrm{FeatureCrop}\left(I,\left\{{Obj}_{i}\right\},F\right)=\left\{p_{key}^{i}\right\},\left\{d_{key}^{i}\right\}$$

Since these are dense points and features, the existing matching algorithm cannot apply for efficiency, and OOPose adopts a dynamic input scale and terminal layer among hierarchical layers. Also, not all objects are equally important, so OOPose selects and matches a core set of objects to be matched by certain heuristics instead of all the objects.

### 3.2. OOPose Framework Overview

**Figure 6:**
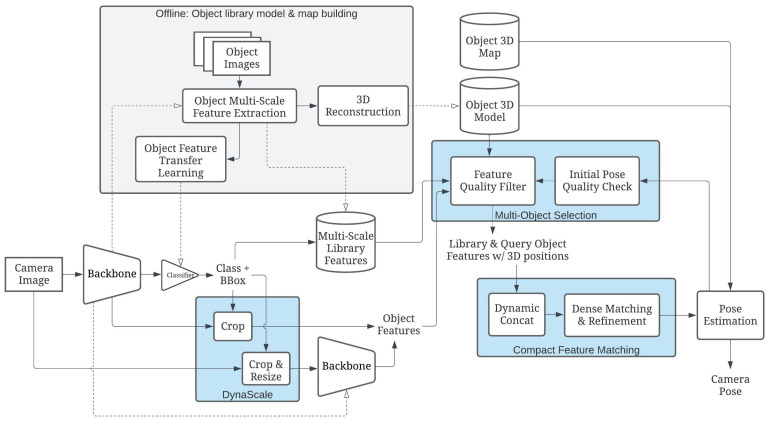
Our proposed object-oriented VBL framework, OOPose, operates as follows: For each camera image input query, we first perform object detection, the results of which are utilized to extract the library region. DynaScale is then employed to crop the feature maps from the backbone of the detector network or resize and extract them if the object size is relatively small. The multi-object selection mechanism selects the most similar pairs of query and library object regions to enhance matching results. Compact feature matching is responsible for finding correspondences between different resolutions of the dense feature maps and refining the original pixel locations for precise localization. Finally, 2D-2D matches between the query and reference images are converted to 2D-3D matches for pose estimation. Multi-scale library features, object 3D models, and map locations are established offline.

Figure 6 provides an overview of the OOPose framework, comprising two stages: offline object learning and map building; and online real-time object-based localization. OOPose offers an innovative approach to localization for IoT systems, enabling an IoT device to determine its location through surrounding unique objects. These unique objects, such as shop signs, brand logos, retail packaging, merchant boxes, posters, or graffiti, exhibit distinct patterns within a confined range of geo-distance without duplicates. Once a few such objects are detected, the device can estimate its pose relative to them and determine its absolute global location by referencing the object map.

During data collection, the framework assumes that objects are stationary and captured by the camera from various distances and viewing angles. Dense features are then extracted at different layers using the object detector backbone for later use in both the transfer-learning phase and the map-building phase.

In the model and map-building phase, features extracted from a sequence of scanned images are utilized to reconstruct object points in the real-world coordinate system. During reconstruction, 2D image points and features are matched across images to establish 2D-2D correspondences. For object 3D models, multiple images capturing the same object are aligned based on the detected bounding box and used to calculate the object’s pose. The accurate scale is recovered using interior sensor priors like Apple ARKit or exterior marker priors like ArUco mapper [76]. The object global position 3D map may be generated through reconstruction or provided via user input.

The framework relies on three databases: the multi-scale object library features, the object 3D models, and the object 3D map. The object library features contain features at several scaled layers extracted from the forward propagation procedure in the neural backbone. The object 3D map is decoupled from the object 3D models to facilitate object movements in global coordinates without altering every 3D point on the object model.

During the online localization phase, the input camera image undergoes object detection, layers of object feature extraction with appropriate sizing, multiple object selection, and object feature matching. Initially, landmark objects are detected from the query input. Subsequently, dense features of the object are extracted from the backbone, either during the detection process if the bounding box size is sufficient or in a second round of backbone network forwarding with the input object region cropped and resized directly from the original camera image. If the number of objects exceeds the requirements, another image processing step to estimate corner feature statistics assists in selecting the subset of objects to be matched. Selected object-dense features are amalgamated into a consistent scale and then matched against corresponding features stored in the library. Matching point pairs are refined and normalized to the original scale by locally searching for similar neighbor features during hierarchical layer traversal. The camera pose is estimated from the point pairs and tested for accuracy to determine the success of the result.

The proposed framework is applicable to various object detectors, and the three key components, DynaScale2, Compact Feature Matching, and multi-object selection, are elaborated upon in the subsequent sections.

#### 3.2.1. DynaScale2: Image Resolution Selection for Object Matching

When features are extracted locally, whether through traditional algorithms or state-of-the-art neural network fused techniques, they encounter limitations in the receptive field’s scope. This limitation arises from the fact that encoded pixel information cannot be matched effectively if they do not share comparable levels of detail. Consequently, object detection aims to identify semantically equivalent regions within the image, thereby setting an upper bound on the space complexity for matching. However, matching images with scale differences remains challenging, as does determining whether an object model or a cropped object region should serve as the library or the query.

To address the first issue, resizing the library image and the query image to a reasonable and similar resolution emerges as a more efficient and effective matching strategy since the shared region of interest has already been identified [14]. However, adjusting the size too much can lead to longer-than-necessary matching times. Conversely, if the size is too small, the features may lack sufficient information for accurate matching.

Secondly, given that object models are typically collected under controlled conditions with minimal noise. In contrast, captured object regions are subject to varying view angles and illumination, it is preferable to match the features of the models with those of the actual scene objects. This approach is more effective than exhaustively enumerating all combinations, as done in DynaScale [14].

Since the neural network is composed of stacked applications of learned filter functions, it creates a higher and higher order of abstraction of images [77] where the features can be utilized as the descriptors [15,42]. The primary question is how to extract the output from those filter functions. According to similar studies on the VGG network [15,42], the consensus is to extract the layers either right after the downsampling layer or the layer second to it. We choose to pick the latter strategy, if applicable, since major lightweight network design jumps into smaller feature maps in the first few layers to avoid a high number of parameter calculations. The computation cost of the VGG network is not feasible for IoT devics, so DynaScale2 extracts the feature maps from MobileNetV3Small [74]-backboned YOLOv5 [75], including the conv3BN or inverted residual layer outputs $F_{k}$ with layer numbers L={k∣k=0,1,3,5,7,9} and their output size being $W_{0}=W/2$, $H_{0}=H/2$, $Ch_{0}=16$, $W_{1}=W/4$, $H_{1}=H/4$, $Ch_{1}=16$, $W_{3}=W/8$, $H_{3}=H/8$, $Ch_{3}=24$, $W_{5}=W_{7}=W/16$, $H_{5}=H_{7}=H/16$, $Ch_{5}=40$, $Ch_{7}=48$, $W_{9}=W/32$, $H_{9}=H/32$, and $Ch_{9}=96$. Note that the additional 1/16-sized layer #7 is not to lose the information caused by the significant gaps between the layers #6 to #8.

**Figure 7:**
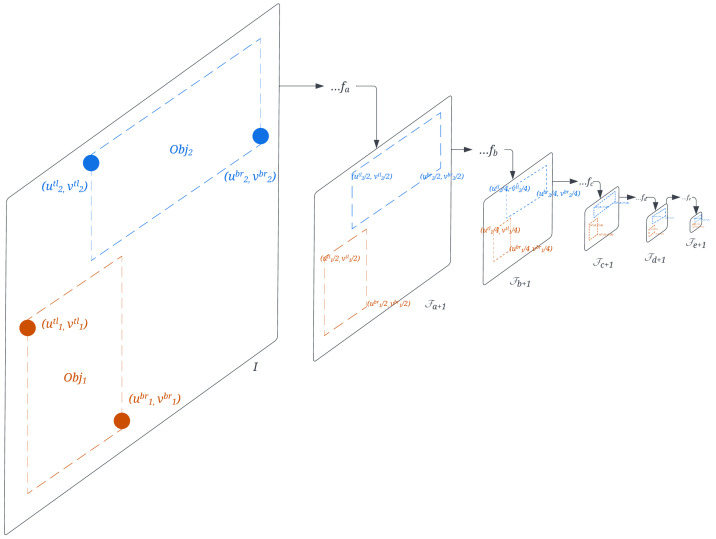
DynaScale2 crops the feature map directly from the detection neural network backbone layers, including the original image layer, the 1/2nd layer, the 1/4th layer, the 1/8th layer, the 1/16th layer, and the 1/32nd layer, based on the coordinates of the objects $Obj_{1}$ and $Obj_{2}$ after downsampling on those layers.

The object detector resizes any input image to a reduced-sized square with a length that is a multiple of 16 or 32, such as 224, 416, 512, or 640 pixels. The minimum size required to match two feature maps is 4×4, corresponding to the smallest intermediate output size of the 1/32-scaled feature maps, further setting the minimum input size to be 128×128. After detecting the bounding boxes, DynaScale2 crops the features directly from the backbone layers, as depicted in Figure 7. The bounding boxes’ top-left and bottom-right corner coordinates are divided by 2 in each operation to down-sample them.

If the object region fed into the detector after transformation is larger than 128×128, which translates to at least a 320×180 pixel object bounding region on a 1280×720 pixel camera image under 512×512 object detection input transformation, DynaScale2 ensures the aspect ratio is maintained to facilitate recovery. Therefore, we propose training the detector on resolutions of 640×360 pixels and 512×288 pixels, respectively, resulting in object region sizes of 256×256 and 320×320, respectively.

Conversely, if the cropped input size of the object regions after transformation is less than 128×128, DynaScale2 extracts the bounding box region from the image at the original resolution to prevent inaccuracies in matching. In such cases, it produces feature maps from the middle layer outputs by reapplying the backbone network.

For optimal localization, it is crucial to match the query input with a resolution resembling the reference one. This can result from either the cropped object detection layers or the reapplied backbone layers. In the former case, only the reference frame needs to be prepared in advance at similar resolutions to avoid additional feature extraction stages. In the latter case, an appropriate input size is essential to minimize computation time, with resolutions of both the reference and the captured query input resized and augmented to multiples of 32, ranging from the minimum size of 128 up to transformed input sizes such as 416, 512, or 640, depending on the network parameters.

#### 3.2.2. Compact Feature Matching

Real-time localization requires accurate and robust point correspondences between matching images within a short period. Sparse features may struggle with changes in viewing angles and distances due to their limited scope. In contrast, dense features offer richer semantics and potentially higher accuracy [15], albeit at a higher computation time cost. By leveraging correspondent object identities and cropped regions, DynaScale2 reduces input size and time costs in object-based localization. However, it is common for relatively small objects to dominate the camera’s field of view, leading DynaScale2 to undergo a second object feature extraction by reapplying the backbone, which introduces a time delay.

Compact feature matching (CFM) aims to dynamically match an object’s dense features across layers extracted from pre-trained network layers, eliminating the need for conventional feature point detection and description on designated small objects. Unlike DFM [15], which relies on fixed layers and inefficiently handles small objects, CFM combines the last few feature layers for matching, enhancing matching accuracy in small object regions.

**Figure 8:**
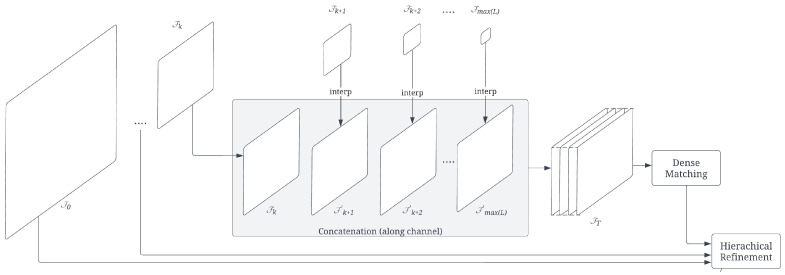
Compact Feature Matching (CFM): it generates the compact terminal feature layer by first interpolation and then concatenation. It proceeds by matching the pair of terminal feature layers and subsequently refines the original pixel location hierarchically through cross-layer nearest neighbor search.

After DynaScale2 obtains intermediate layers $F_{k}$ from the network backbone, CFM creates a semantically dense representation of the image by interpolating higher-level semantic layers and aggregating them to generate the combined terminal layer feature map, $F_{T}$. Similar to the matching stated in DFM [15], the initial matching occurs at the terminal layer with the layer number T∈L where T=max(L), which means the deepest layer which contains the most semantic information. However, due to the varying size of the cropped object region, especially the smaller ones, we instead decide the terminal layer resolution based on the input size to ensure the minimum resolution of the terminal layer should be at least 4 × 4, so $T=\max(L^{\prime })$ and $L^{\prime }=\left\{k|k\in L\;\mathrm{and}\;W_{k}\geq 4\;\mathrm{and}\;H_{k}\geq 4\right\}$. Each of the extracted layers smaller than the resolution is upsampled by the multiples of 2 and interpolated to match the required resolution and then concatenated along the channel axis to become the new combined terminal layer $F_{T}=\mathrm{concat}\left(F_{k}^{\prime },F_{k+1}^{\prime },\cdots ,F_{\max(L)}^{\prime }\right)$ where $k=\min(L-L^{\prime })$ and $F_{k}^{\prime }=\mathrm{interp}\left(F_{k},\left(W_{k}\times 2^{T-k},H_{k}\times 2^{T-k}\right)\right)\in R^{W_{T}\times H_{T}\times Ch_{k}}$. The concatenation function, concat, refers to the appending of a 3D tensor vector along the channel so that $F_{T}\in R^{W_{T}\times H_{T}\times \sum_{i=k}^{\max(L)}Ch_{i}}$. The interpolation function, interp, upsamples the smaller input $F_{k}$ into a larger feature map $F_{k}^{\prime }$ at a size of $W_{T}\times H_{T}$. Figure 8 shows the terminal layer aggregation from interpolation and concatenation of the extracted object detection feature maps. The resolution of the terminal layer $W_{T}\times H_{T}$ cannot be either too large or too small because the larger the size, the more expensive the matching costs, and the smaller the size, the higher the ambiguity contained in each point of the feature map, which impedes the matching accuracy.

The nearest-neighbor-based dense matching is performed on the two combined terminal layers, $F_{T}^{A}$ and $F_{T}^{B}$, computed from the two cropped object image inputs $I^{A}$ and $I^{B}$ to first find the dense correspondence set $C=\left\{c_{i,j}\right\}=\left\{\left(p_{i}^{A},p_{j}^{B}\right)\right\}$, where $\left|C\right|=W_{T}^{A}\times H_{T}^{A}$, $i=\{1,\ldots ,W_{T}^{A}\times H_{T}^{A}\}$, $j=\{1,\ldots ,W_{T}^{B}\times H_{T}^{B}\}$, and $\left(W_{T}^{A},H_{T}^{A}\right),\left(W_{T}^{B},H_{T}^{B}\right)$ are the widths and heights of feature maps $F_{T}^{A}$ and $F_{T}^{B}$ where the matched points $p_{i}^{A}$ and $p_{j}^{B}$ extracted, respectively. Among the correspondence set, the correspondences that have the mutually nearest distance and pass the Lowe’s ratio test [17] will be considered inliers.

Afterward, hierarchical refinement [15] recovers the positional points at the immediate previous layer k−1 from the matched points at the current layer *k* by matching the features at layer *k* to the other features at the neighborhood positions within the same receptive field on the next level feature map k−1. Because the resolution of the layers is twice different between the consecutive extracted layers, the receptive field contains the point features with twice the coordinates and those points with the location one-pixel right and bottom-right and bottom neighborhood point features, and the refinement can be restricted to comparing each upsampled feature at a level *k* to those 4 neighboring features at the next larger feature map k−1. Besides, some network backbones, such as MobileNet [74] series, do not have the low-level convolution layers with size $W_{0}=W$, $H_{0}=H$ as in VGG-series [73], for the need of speed. In this case, CFM includes the original 3-channel RGB image as the final layer $F_{0}$ for the hierarchical refinement to recover the matched pixel position at the original scale of the input image.

#### 3.2.3. Multi-Object Selection and Pose Estimation

The basis of matching between library and query images involves computing the similarity scores between two sets of features, which requires calculating the inner product between the two vectors. For matching object regions, the computational complexity is O(kmnl), where *k* is the number of objects, *m* is the average number of object features in the library model, *n* is the average number of object features in the query region, and *l* is the bit length of the feature descriptors. Although reducing the matching cost to specific regions with object bounding boxes compared to features of the entire image is advantageous, excessive numbers of objects can inflate the matching cost to a level similar to that of matching the whole image, offering no real-time processing benefits.

Furthermore, the size and number of visible objects depend on their distances from the camera. Closer objects cover more area on the camera frame, with clear feature points for comparison. In comparison, objects further away result in more objects being captured, but with blurred and indistinguishable feature points, compounded by variations in illumination, weather, and motion blur. Including all detected objects may only improve accuracy while significantly increasing computation costs. Hence, matching fewer but quality-resembled objects, where quality is measured by the quantity of low-level, reliable, and accountable features, can decrease the required matching time.

The Features from Accelerated Segment Test (FAST) [19] is a high-speed point detection algorithm designed for real-time feature point detection by comparing grayscale pixel values along a circle (typically with a 3-pixel radius, totaling 16 pixels on the circle) around a candidate point. If a certain number of contiguous pixels (usually 12 pixels) have higher or lower intensities than the value of the candidate point, the candidate is identified as a detected FAST feature point. The number of FAST keypoints represents the image quality, favoring contrast around the radius of the keypoints, particularly in corner patterns. Changes in illumination, misclassification due to incorrect detection, motion blur, or partial obstruction by other objects can dramatically alter the contrast signature, indicating quality changes. Larger discrepancies in the number of FAST points between library and query object regions degrade matching algorithm performance.

To prioritize objects, we first detect FAST keypoints and compare the difference between the number of keypoints in each object’s library and the corresponding query region. Before entering the region feature-map pairs into CFM, the multi-object selection module ranks the top-*k* objects based on the resemblance in quality of the two object regions, determined by the closest number of FAST keypoints each contains. The selection process is outlined in Algorithm 1.

After matching, the final step is pose estimation, which computes the camera location based on the available library models. Two types of pose estimation are currently supported: one for planar objects and the other for 3D objects. Regardless of the type of objects captured by the camera, accurate pose estimation requires the correct mapping between pixel locations and established 3D coordinates in the map.
**Algorithm 1** Multi-Object Selection for Matching.**Require:** $N_{k},I^{Q},{\mathrm{DB}}^{L},f$         ▹ # of Objects to pick, Query img, Library & NN
**Ensure:** $F^{Q^{\prime }},F^{L^{\prime }}$         ▹ Object query & library features selected for matching
 1: ${\mathrm{Obj}}^{Q},F^{Q}\leftarrow \mathrm{DYNASCALE}2\left(f,I^{Q}\right)$         ▹ Detect objects & Extract features
 2: $F^{L},S\leftarrow \phi$
 3: **for** 
$\left(Obj^{Q},F^{Q}\right)\mathrm{in}\left({\mathrm{Obj}}^{Q},F^{Q}\right)$ 
**do**
 4:    ${NF}^{Q}\leftarrow \#$FAST$\left(F_{0}^{Q}\right)$         ▹ Get #FAST from RGB of object region
 5:    $F^{L},{NF}^{L}\leftarrow \mathrm{GETFEATURES}\left({\mathrm{DB}}^{L},\mathrm{GETCLASSID}\left(Obj^{Q}\right)\right)$
 6:    $S\leftarrow S+\{\left|{NF}^{Q}-{NF}^{L}\right|\}$
 7:    $F^{L}\leftarrow F^{L}+\left\{F^{L}\right\}$
 8: **end for**
 9: $S^{\prime }\leftarrow \left\{s^{\prime }\in S|s^{\prime }\leq \min\left(t\in S\;\mathrm{such}\;\mathrm{that}\;\#\left\{s\in S|t\leq s\right\}=N_{k}\right)\right\}$
10: $F^{Q^{\prime }},F^{L^{\prime }}\leftarrow \phi$
11: **for** 
$s\mathrm{in}S^{\prime }$
**do**
12:    i←index(s,S)
13:    $F^{Q^{\prime }}\leftarrow F^{Q^{\prime }}+\left\{F_{i}^{Q}\right\}$
14:    $F^{L^{\prime }}\leftarrow F^{L^{\prime }}+\left\{F_{i}^{L}\right\}$
15: **end for**


For planar objects, matched pairs of keypoints on the plane are converted into the four points bounding the surface’s four corners. Assuming the matched points are coplanar, a homography matrix H can be computed in a few milliseconds for this 2D-to-2D motion using a minimum of four pairs of matched 2D keypoints. The camera pose can then be estimated from the surface corners. Given correspondence results $C=\left(p_{i}^{A},p_{j}^{B}\right)$, where $M^{A},M^{B}$ represent the homography mapping relationship from points on one plane to the other in matrix form (where points $p^{B}i$, $p^{A}i$, where 1≤i≤|C|, are row vectors of the matrix indicating object points), it can be represented by the following formula:

$$M^{B}=\left[\begin{array}{ccc} — & p_{1}^{B} & —\\ — & p_{2}^{B} & —\\ & \vdots & \\ — & p_{|C|}^{B} & — \end{array}\right]=H\cdot M^{A}=H\cdot \left[\begin{array}{ccc} — & p_{1}^{A} & —\\ — & p_{2}^{A} & —\\ & \vdots & \\ — & p_{|C|}^{A} & — \end{array}\right]$$

While a homography matrix describes the mapping from one 2D planar object to another, we evaluate the camera pose from the four corners of the plane, which are projected by the matrix from the 3D coordinates of those corners stored in the object model. We choose not to estimate 3D locations directly from all the 2D planar feature points for two reasons. First, collecting and localizing the 3D coordinates of non-corner points on the surface beforehand is crucial to avoid bias in the following camera pose estimation due to measurement noise. These coordinates must be measured with higher accuracy than the camera deployed during runtime and may need interpolation from finer specifications or additional input, such as the dimensions of the object silhouette points or data from a high-resolution depth sensor, which often requires post-optimization and triangulation from other technologies like laser-based motion capture [78]. Second, we can treat the calculation as a filter by checking certain properties of the homography transformation results to ensure the quality of matching and rule out incorrect poses, as proposed in [14].

The second type of more general 3D object presents a greater challenge, as the shape and feature distinctiveness vary widely. For such complex-shaped objects, reconstructing the 3D library model requires a sequential 2D image scan of the objects, with or without depth information, as described in [8,16,61].

**Figure 9:**
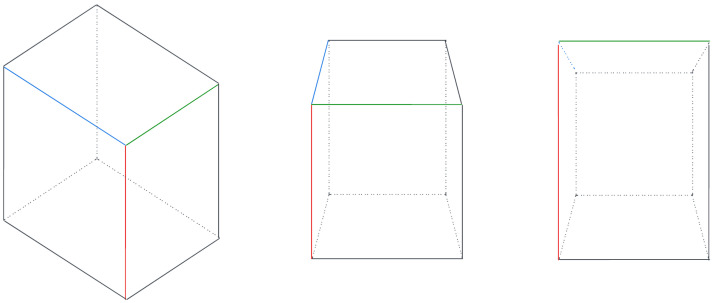
Combinations of the 6-face model for 3D objects: A 3D object can be divided into six viewing faces orthogonal. When a detected object’s 2D box is identified, there may be scenarios where three faces, two faces, or one face of the object are facing the camera. Initially, all six candidates need to be considered. Once the facing view is determined, the possibilities are reduced to two candidates for three faces and four candidates for two faces.

During runtime, the initial views of the object that the camera faces are unknown, as illustrated in Figure 9. Identifying the view requires locating the library features first, which can be accomplished through various methods such as ID detection of artificial tags [13], high similarity scores in global feature descriptor spaces [8], or matching an entire set of fused local 2D object features via graph algorithms [61]. The precision with which the library features are identified determines the accuracy of the matching outcome for pose estimation. However, previous methods relied on the distinctiveness of repeatable query and library features and aimed to select the most characteristic points for matching. Achieving such goals is challenging and often not feasible due to symmetrical, repeated, or noisy patterns under different angles and illuminations in the physical world. Therefore, the pose outcome after matching should be the primary evaluation criterion, rather than focusing solely on the feature points and descriptors.

We propose Algorithm 2 to estimate the initial camera pose from surface models of a 3D object by reusing features extracted from the neural network backbone and filtering out incorrect poses.

Given the various possible views in which an object could be captured, determining which sides of the object face the camera each time it is detected incurs significant computational costs. To mitigate this overhead, successfully matched objects are integrated into the library model with estimated 3D coordinates for future matching. Consequently, in subsequent frames, matching between the registered real-scene object library model and the captured object image region becomes faster and more straightforward as their features and scales closely resemble those of the pre-built library.

Using the precise dimensions of the library objects, we compute the camera transformation matrix relative to the captured objects by applying the Perspective-n-Point (PnP) algorithm to matched pairs of 2D corner pixel locations and their corresponding 3D model corners. We employ iterative Levenberg-Marquardt optimization to minimize re-projection errors of image pixels to 3D coordinates [79]. However, practical constraints result in inevitable errors among the matched pairs, leading to the failure of direct matrix calculation. Therefore, filtering out outliers and minimizing the overall transformation error among the remaining inlier-matched points is necessary. We employ a RANSAC-based method [80], which efficiently estimates a model by identifying the largest inlier subset within an acceptable error threshold. The process involves selecting a subset of randomly chosen point pairs, calculating the projection or transformation model, categorizing other points as inliers or outliers based on a given re-projection error threshold, and iteratively refining the model until the maximum number of iterations is reached. The final model is determined based on the subset with the maximum number of members, representing the consensus among random samples.
**Algorithm 2** Initial Object Pose Quality Check.**Require:** $Obj^{Q},F^{Q},{\mathrm{DB}}^{L}$▹ N/A
**Ensure:** N/A▹ N/A
 1: **if** $Obj^{Q}$ is not the first-time detected **then**
 2:   **return**
 3: **end if**
 4: $FACES\leftarrow \mathrm{Enumerate}\;\mathrm{faces}\;\mathrm{from}\;\mathrm{model}({\mathrm{DB}}^{L},Obj^{Q})$
 5: $MASK^{Q}\leftarrow \mathrm{Generate}\;\mathrm{all}-\mathrm{true}\;\mathrm{mask}\left(F^{Q}\right)$
 6: VALIDS←ϕ
 7: **while** FACES is not empty **do**
 8:    fid←FACES.pop()
 9:    $F^{L},MASK^{L},x^{L}\leftarrow \mathrm{GETFACE}\left({\mathrm{DB}}^{L},\mathrm{GETCLASSID}\left(Obj^{Q}\right),fid\right)$
10:    $p^{L},p^{Q}\leftarrow \mathrm{CFM}\left(F^{L},F^{Q},MASK^{L},MASK^{Q}\right)$
11:    **if** QUALITYTEST($p^{L},p^{Q}$) is not passed **then**
12:       **continue**
13:    **end if**
14:    VALIDS.add(fid)
15:    FACES←ϕ
16:    T←ESTIMATEPOSE($x^{L},p^{L},p^{Q}$)
17:    $p^{\prime }\leftarrow$ project all endpoints of faces in $x^{L}$ by *T*
18:    **if** VALIDS.size()=1 **then**
19:       **for** nfid
**in** neighboring face IDs of fid **do**
20:           **if** nfid **in** VALIDS **or** any of its endpoints **in** area of fid **then**
21:            **continue**
22:           **end if**
23:           FACES.add(nfid)
24:           $MASK^{Q}\leftarrow$mask out the area of nfid in $MASK^{Q}$
25:       **end for**
26:    **end if**
27: **end while**


## 4. Ablation Study

In this section, we evaluate the performance of our framework and assess how each module contributes to overall localization accuracy and speed through a series of studies. Firstly, we demonstrate that compact feature matching, executed on the CPU, outperforms the state-of-the-art DFM [15] and conventional ORB-based techniques regarding both speed and accuracy in camera pose estimation. Secondly, we compare compact feature matching with popular real-time ORB feature matching, both with and without input scale adjustment, to highlight differences in accuracy. Thirdly, we examine how multi-object selection can achieve balanced results and expedite the overall process. Lastly, we conduct runtime analyses on various IoT devices.

The hardware used for testing include: (1) an Intel i7-8650U CPU with 16GB memory priced at USD$800, (2) a Jetson Nano Developer Kit with 4GB memory priced at USD$150, and (3) a Jetson Xavier NX reComputer J2021 with 8GB memory priced at USD$600. The software is implemented using the OpenCV3-Python3 library on Ubuntu 18.04 64-bit OS.

We utilized three datasets in our studies: the JavaCity dataset, the BoxPose dataset, and the VP dataset. The JavaCity dataset comprises 480 video frames recorded at 30 FPS in the outdoor JavaCity Cafe dining area using an Apple iPad Pro equipped with low-cost LIDAR. The ground truth pose for each frame was calculated using the ORBSLAM3-RGBD system with full loop-closing video. It contains various unique solid objects, including shop signs, refrigerators with brand signs, poster ads, food/drink boxes, trash cans, and bags.

The BoxPose dataset consists of six frames capturing a single box object, with an ArUco tag on each of its six faces serving as ground truth for pose. Each frame varies in viewing angle and distance to the object.

**Figure 10:**
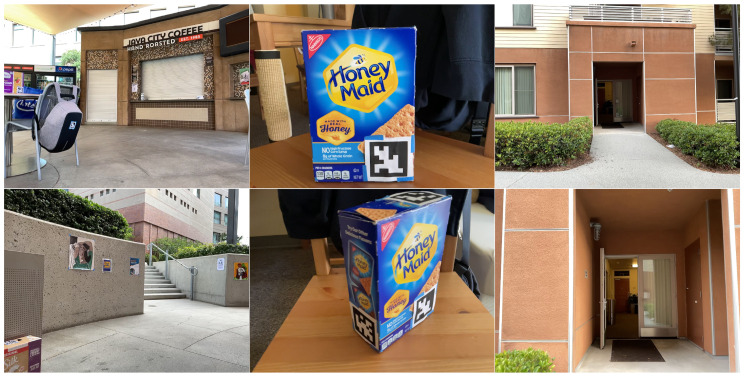
Examples of the JavaCity, BoxPose, and VP dataset (From left to right columns).

The VP dataset, recorded with an iPad device and utilizing ORBSLAM3-RGBD for ground truth, consists of 700 frames captured outdoors on the sidewalk leading to a 1F apartment building room. It features a different set of unique objects, including bushes, entrance doors, poster ads, lamps, plants, chairs, and desks. Objects are first detected using an objectness detector to identify potential static entities and then tracked using a correlation-based object tracker with high Intersection over Union (IoU) and consistent bounding box sizes between consecutive frames to ensure object ID consistency. Examples of each dataset are illustrated in Figure 10.

### 4.1. Metrics

Four metrics are used for comparison: accuracy, speed, robustness, and price. Accuracy refers to the pose error between the predicted position and the ground truth. Speed is measured in computation time, typically in seconds. Robustness indicates the number of valid frames with output pose errors smaller than 50 cm. Different applications prioritize these objectives differently, but none can be overemphasized as the localization software runs on IoT devices with limited resources. Any changes in the priority of one metric result in adverse effects on the other metrics. For instance, prioritizing higher accuracy may increase model complexity or input size, thus increasing running time. Similarly, using more capable hardware uniformly improves speed, and accuracy increases with larger deployed models, but this also affects the price.

To compare different hardware options, it is essential to consider the marginal gain of accuracy, speed, robustness, and computational power per unit price. This measure indicates how much performance can be gained with an additional unit of budget.

### 4.2. Backbone for Feature Matching

VGG-19 [73], as adopted in DFM [15], serves as the baseline for dense feature matching due to its 16 feature extraction convolution layers, totaling 19.6 FLOPS. While it offers abundant features across channels ranging from 64 to 512, adding more layers to the VGG network exacerbates the vanishing gradient problem during training. To address this, skip connections, introduced in ResNet [81], complement the input identity into the output, facilitating gradient flow during backpropagation without diminishing to zero. MobileNet [74] further optimizes computational efficiency through depthwise separable convolutions, ResNet-like fast connections, squeeze and excitation layers, and network architecture search. These networks share a similar design of reducing feature dimensions block by block, enabling extraction of outputs from middle layers immediately after downsampling layers, as utilized in DFM [15].

Table 2 compares dense feature matching on the JavaCity dataset using off-the-shelf neural network detector backbones: VGG-19, MobileNetV3-Small, MobileNetV3-Large, ResNet-50, and ResNet-101. VGG-19 yields the highest number of valid frames with reasonable accuracy, averaging 18.3 cm. However, it incurs the longest execution time for a single matching, offering less than 5 FPS on the CPU, rendering it unsuitable for real-time applications. ResNet-50 and ResNet-101 reduce the time but struggle to produce valid poses due to the indistinctiveness of features consistently, compounded by the skip connection’s inclusion of identity into the output, leading to unavoidable isolation and increased time cost. Both MobileNetV3-Small and MobileNetV3-Large significantly enhance speed by over five times, with an acceptable decrease in accuracy and robustness around 20% for MobileNetV3-Small and 10% for MobileNetV3-Large. Of the two, MobileNetV3-Large demonstrates superior accuracy, with an average pose error of 20.6 cm and 85.4% of its frame output poses being valid while achieving a real-time performance of 25 FPS. Consequently, it is chosen as the feature extraction backbone for our object-oriented framework, encompassing the DynaScale2 dense feature extraction and subsequent CFM dense matching modules.

### 4.3. Input Scale Change for Real-Time Performance

The input scale plays a significant role in determining the number of parameters computed by convolution filters. As discussed in the DynaScale2 Section 3.2.1, larger images require more computation time, increasing quadratically. Conversely, images should not be too small, as features mixed in a single frame frustrate similarity computation between library and query image features, leading to unstable matching and lower localization accuracy and robustness. To evaluate real-time performance under input scale changes, we compare our CFMs with and without DynaScale2, adjusting the input to 416 pixels, against the representative ORB [20] 2k sparse feature points matching on the BoxPose dataset. Each detected region is matched against the corresponding original library with the same object ID. Table 3 presents the pose error and the time taken to compute features, match them, and estimate the pose.

From Table 3, we observe that matching at a standard scale poses challenges for both methods, as each feature can only encode surrounding information within its receptive field. If viewing angles and distances vary significantly, features extracted from object regions contain contrasting details. Dense matching avoids pose estimation failure by comparing all points, but it cannot be processed in real-time, achieving only 1.3 FPS, while ORB runs at 10 FPS but fails to match consistently, with 33% of frames failing to localize.

With DynaScale2, both methods achieve more accurate localization and faster speeds, reaching 11 FPS on the CPU, as matching becomes easier in reasonably smaller and similar regions, yielding fewer but more alike features. Our method demonstrates even better average accuracy, with a 32% increase, without suffering from the heavy load of conventional dense matching. This showcases the dynamic input scale change and selective combination of feature layers from intermediate layers with different dimensions, proving that DynaScale2 and CFM enhance pose accuracy and robustness without sacrificing computation speed.

### 4.4. Multi-Object Selection

**Figure 11:**
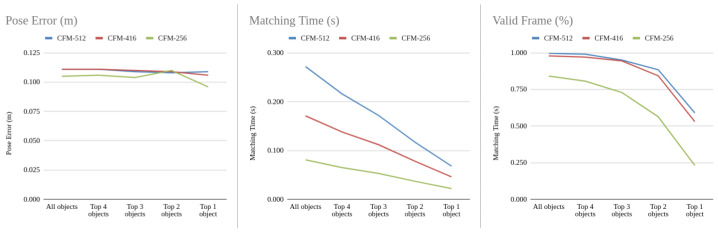
Comparison of pose error, matching time, and the robustness between different resized inputs and a various number of objects selected.

The objective of multi-object selection is to reduce computation time spent on matching by selecting a subset of objects based on their quality similarity. In Figure 11, we test the selection algorithm on the VP dataset to evaluate its performance under different top-*k* selections (from k=1 to 4) or select all objects.

From the pose errors, we observe minimal differences between the number of objects selected, all capable of localizing the camera with errors ranging from 10–12.5 cm. As expected, matching time increases with the number of selected objects. Regarding robustness, the number of valid frames decreases non-linearly as the number of objects decreases.

The computational burden induced by input scale changes is significant. Comparing CFM-512 and CFM-416 to CFM-256, matching time almost doubles and triples, respectively, increasing from less than 100 ms to roughly 200 ms or 300 ms when processing all objects. There is also a disparity in robustness, notably with CFM-256 having the lowest number of valid frames among the three, with 20% fewer.

To achieve practical speeds of around or greater than 10FPS, CFM-256 with top-4 objects or CFM-416 with top-3 objects are recommended. The former offers higher speed, while the latter is more robust. Low latency is preferred over robustness when the robot is in motion as scenes change rapidly. During relocation, however, robustness is prioritized over speed to increase the chances of success within real-time constraints.

### 4.5. Execution Time of First-Time Object Detection

The execution time from image input to pose output comprises several steps: object detection, the impact of two stages of DynaScale2, and multi-object selection. Previous studies did not consider object detection time. Still, our system requires detecting the ID of any new unique object the first time it appears, as it will be tracked later to reduce execution time. We evaluate the performance of object detection between YOLOv5s and YOLOv5s with the MobileNetv3 backbone on different IoT hardware.

Table 4 presents the conventional time benchmarks of the YOLOv5s framework with its customized backbone and the MobileNetV3 backbone across different input image sizes (640×640 and 512×512) and various hardware configurations. “Intel-CPU-pytorch” denotes running the network models without optimization on an Intel CPU, while “Intel-CPU-ONNX” does so with hardware compression optimization. “JN-pytorch” and “JX-pytorch” are similar setups but with models accelerated on Jetson Nano and Jetson Xavier GPU, respectively. “JN-trt” and “JN-trt-FP16” represent Nvidia tensor RT optimization versions of the models without and with floating point precision reduced to 16 bits, respectively. The MobileNetV3 backbone demonstrates 3 to 40 ms less time for object detection, indicating better real-time performance.

Regarding hardware choices, the Jetson Xavier outperforms the Intel-CPU regarding marginal costs. Although the Xavier costs four times more than the Nano, it achieves two to three times faster detection times. In contrast, the Intel-CPU setup is 5.3 times more expensive than the Jetson Nano but only provides twice the speed improvement. While the budget increase may not justify the performance difference between the Jetson hardware, when considering the detection time added to the matching time costs discussed in previous sections for achieving 10FPS or more, the Jetson Xavier exhibits fewer time delays (40–50 ms) compared to the Jetson Nano (80–120 ms).

## 5. Conclusions

In this paper, we introduced the OOPose framework, a pioneering solution for visual-based localization in urban environments, enabling IoT devices to achieve self-localization with just a camera. The core innovation lies in leveraging known 3D maps of objects, allowing camera-equipped devices to swiftly and accurately determine their location. By adopting adaptive scaling techniques, utilizing off-the-shelf neural network features dynamically, and selecting the most similar object candidates, OOPose achieves real-time, accurate, and robust performance without additional networks or feature detectors. This low-cost method applies to various mobile devices, including smartphones, tablets, and small robots, making it a practical localization solution for diverse scenarios such as service robots or augmented reality.

Future research could extend the framework to handle diverse input and output modalities for other image processing fields. The applicability of this method extends beyond the realms of the Internet of Things, finding potential applications in various physical areas related to image processing, such as surveillance, industrial automation, and medical imaging.

## Figures and Tables

**Table 1:** Comparison of visual-based localization methods concerning the precision, the speed, the object content inclusion, the actual scale, the processing stages, the input size adjustment, the multi-object selection, and the type of object used.

Method		Precision	Speed	Object Content	Actual Scale	Stages	Dynamic Adjust Input Image Size	Multi-Object Selection For Efficient Matching	Object Type
Direct Image (VPL [7])		Low	O(library size)	N/A	No	Local feature detection + description + global clustering	No	No, one global descriptor per image	N/A
Indirect Pixels (mono-ORBSLAM3 [10])		Med-High	Medium	N/A	No	Local feature detection + description	No	No, Pixel descriptors from whole image	N/A
Object-based	ArUco [13]	High	High	Square-bordered encoded blocks	Yes	Corner detection + cell decoding	No	No, match all squares	2D
	Picpose [12]	High	Med-High	Any patterned plannar picture	Yes	Object detection + local feature detection + description	No	No, match all object regions	2D
	OOPose	High	Med-High	Any patterned object	Yes	Object detection + dense feature crop	Yes	Yes	3D

**Table 2:** Comparison between VGG19, MobileNetV3-Small, MobileNetV3-Large, ResNet50, ResNet101, on the performance of pose errors, robustness, and computation time costs along with the percentage of difference of each metric to those of the VGG-19. The best results are in bold font.

	VGG19	MNV3-S	MNV3-L	ResNet50	ResNet101
Pose Error (m)	0.183	0.232 (+26.7%)	0.206 (+12.5%)	0.155 (−15.3%)	0.168 (**−8.2%**)
Valid Frames (%)	96.0	73.6 (−22.4%)	85.4 (**−10.6%**)	39.4 (−56.6%)	45.4 (−50.6%)
Time (s)	0.219	0.034 (**−84.5%**)	0.040 (**−84.4%**)	0.081 (−63.0%)	0.156 (−28.7%)

**Table 3:** Comparison of the object-oriented localization performance between dense feature CFM and sparse feature ORB and their improvement with DynaScale2 applied.

	CFM-416	Sparse ORB-2k
DynaScale2	Avg error: 1.06 cm (100%) Min error: 0.5 cm Max error: 2.3 cm Avg Time: 86 ms (100%)	Avg err: 1.4 cm (+32.1%) Min error: 0.3 cm Max error: 4.6 cm Avg Time: 87 ms (+1.16%)
Normal Scale	Avg error: 2.56 cm (+141%) Min error: 1.0 cm Max error: 7.3 cm Avg Time: 747 ms (+768%)	33% frames failed (N/A) Min error: 0.3 cm Max error: 0.7 cm Avg Time: 92 ms (+6.97%)

**Table 4:** Comparison of the object detection execution time under choices of input sizes and IoT hardwares.

	640x640		512x512	
	**YOLOv5s**	**YOLOv5s-mv3**	**YOLOv5s**	**YOLOv5s-mv3**
CPU-pytorch	103.76 ms (100%)	63.21 ms (100%)	77.32 ms (100%)	45.31 ms (100%)
CPU-ONNX	78.88 ms	55.41 ms	52.40 ms	33.56 ms
JN-pytorch	170 ms (+60%)	119.6 ms (+20%)	111.86 ms (+40%)	80.82 ms (+80%)
JN-trt	120 ms	85.4 ms	74.43 ms	73.79 ms
JN-trt-FP16	82 ms	67.4 ms	54.46 ms	N/A
JX-pytorch	49.61 ms (−53%)	44.58 ms (−29%)	47.83 ms (−38%)	44.74 ms (−1%)

## Data Availability

Data are contained within the article.

## Abbreviations

The following abbreviations are used in this manuscript:

OOPose	Object-Oriented Pose
AR	Augmented Reality
IoT	Internet of Things
VBL	Visual-Based Localization
GPS	Global Positioning System
CPU	Central Processing Unit
GPU	Graphic Processing Unit
CAD	Computer-Aided Design
ORB	Oriented FAST and Rotated BRIEF
FAST	Features from Accelerated Segment Test
CFM	Compact Feature Matching
FLOPS	FLoating-point Operations Per Second
